# Chronic constipation that resulted in fecal impaction and colon perforation: A case report

**DOI:** 10.1097/MD.0000000000030206

**Published:** 2022-08-26

**Authors:** Ahmed Alburakan, Aljoharah Alshunaifi, Razan AlRabah, Sulaiman Alshammari, Saleh Alnasser, Thamer Nouh

**Affiliations:** a Trauma and Acute Care Surgery Unit, Department of Surgery, College of Medicine, King Saud University, Riyadh, Saudi Arabia; b College of Medicine, King Saud University, Riyadh, Saudi Arabia; c Department of Surgery, College of Medicine, King Saud University, Riyadh, Saudi Arabia; d Thoracic Surgery Unit, Surgery Department, King Saud University Medical City, King Saud University, Riyadh, Saudi Arabia.

**Keywords:** gastrointestinal motility disorders, constipation, dysphagia, perforation

## Abstract

Gastrointestinal (GI) motility disorders represent a set of variable presentations caused by an abnormal functioning enteric neuromusculature. Any part of the GI tract can be affected, and depending on the organ involved, the patient presentation will differ.

**Patient concerns:**

A 26-years old female who had a history of laparoscopic Heller myotomy 15 years ago for progressive dysphagia. She presented with peritonitis and sigmoid colon perforation secondary to severe chronic constipation. Later after undergoing Hartman procedure, she continued to have significant constipation. In addition, she reported progressive dysphagia and regurgitation to both solids and liquids.

**Diagnosis:**

An esophageal manometry revealed Achalasia type 3, and stomach motility nuclear study showed mild delay in gastric emptying.

**Interventions:**

Initially, Hartmann procedure was performed. Afterward, we performed a reversal of Hartman, robotic redo of Heller myotomy, and Dor fundoplication was performed.

**Outcomes:**

The patient had an uneventful postoperative course and was discharged in good condition.

**Lessons:**

Our case highlights an unusual presentation of GI motility disorder resulting in peritonitis from sigmoid colon perforation. Early recognition and prompt treatment of GI motility disorders are essential to avoid severe complications.

## 1. Introduction

Gastrointestinal (GI) motility disorders are a group of conditions characterized by impairment in the enteric neuromusculature. This may involve any part of the GI tract from the oropharynx to the anorectum.^[1]^ Patients can have different presentations according to the organ involved, such as achalasia, nonachalasia esophageal motility disorders, gastroparesis, chronic intestinal pseudo-obstruction, irritable bowel syndrome, and chronic constipation.^[2]^ The underlying pathology is functional in these conditions, and no organic cause can be identified. Conversely, many organic GI disorders can present with a similar presentation.^[1]^ We describe a case of chronic constipation that resulted in fecal impaction and colon perforation. Upon investigation, the patient had achalasia and delayed gastric emptying.

## 2. Case report

A 26-year-old female presented to the emergency department complaining of abdominal pain in the right lower quadrant and suprapubic area for 3 days. The patient also reported nausea and watery diarrhea without mucus or blood. She had a similar episode 3 months before presentation, which was managed with analgesia. The patient did not have any fever, night sweating, or weight loss history. There was no history of travel to parasite-endemic areas, and the patient denied any history of trauma. She had a remote history of laparoscopic Heller myotomy 15 years ago for progressive dysphagia. The rest of the medical history was unremarkable. During the physical examination, the patient was tachycardic and in moderate pain. There was abdominal tenderness over the suprapubic area and positive rebound tenderness in the right lower quadrant. Routine laboratory tests, including complete blood count, liver function test, and renal profile, have been performed and were unremarkable. Erect and supine abdomen x-rays were unremarkable as well.

**Figure 1.**
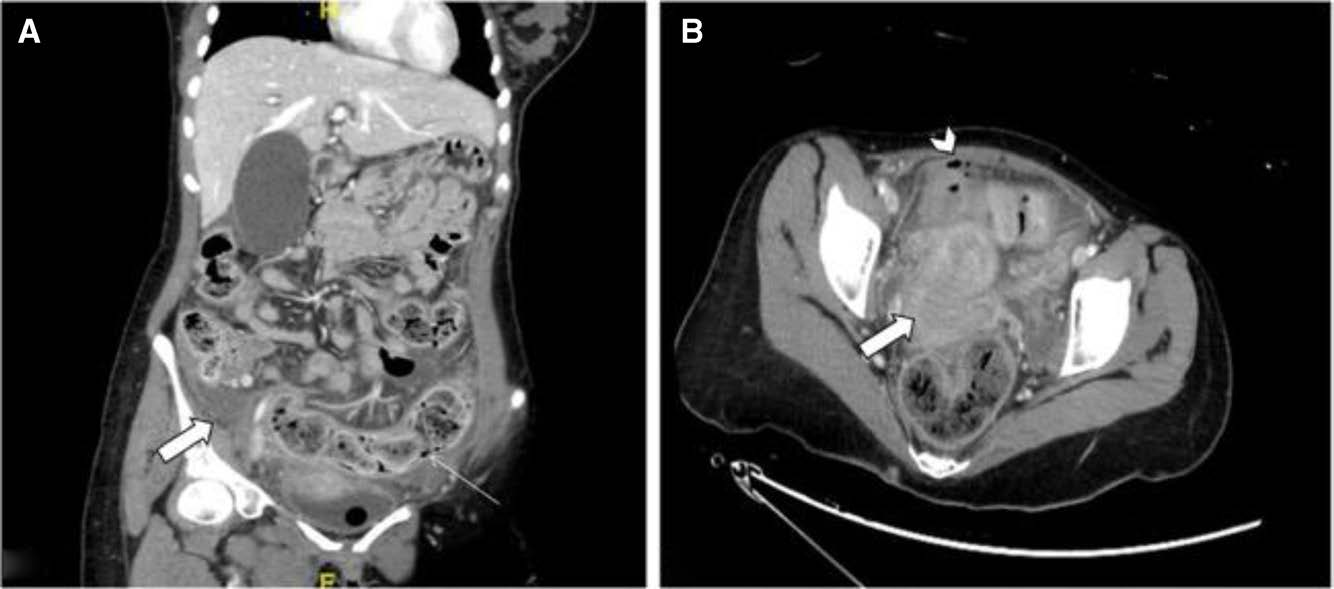
CT abdomen images: (A) coronal view with findings of sigmoid colon loaded with stool (small arrow) and a small amount of abdominopelvic fluid (large arrow). (B) axial view with results of pelvic fluid (large arrow) and foci of pneumoperitoneum (arrowhead).

Computed tomography (CT) abdomen and pelvis with Intravenous (IV) contrast were performed, reviewed, and reported by a senior radiologist. Her CT scan revealed the presence of pneumoperitoneum, diffuse enhancement of the peritoneal reflection, and a moderate amount of abdominal pelvic ascites (Fig. 1). There was no definite site of perforation. The gallbladder was diffusely distended with mild intrahepatic biliary dilatation. The overall findings were highly suggestive of a perforated viscus.

The plan was to proceed with diagnostic laparoscopy, possible laparotomy, bowel resection, and colostomy. Written informed consent was obtained from the patient.

**Figure 2.**
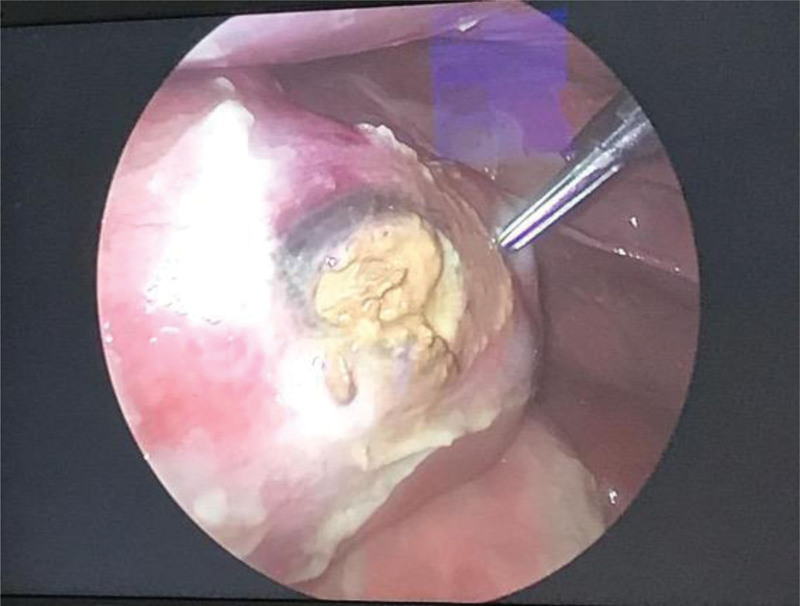
Photo of laparoscopy screen showed sigmoid colon perforation secondary to hard stool impaction with ischemic changes of the perforation edges.

The patient underwent laparoscopic exploration, where she was found to have a fecal mass causing perforation and pus collection. (Fig. 2). An open Hartmann procedure was performed, large amounts of fecal matter were removed, and the sigmoid was resected involving the perforation site. The pathology department received a segment of large bowel measuring 12 cm in length and 2.5 cm in diameter. Gross examination showed a perforation measuring 2.5 cm in maximum dimension. The serosal surface was congested and covered by an exudate. Sectioning of the specimen revealed focal ulceration and macroscopic perforation.

The patient was admitted to the intensive care unit postoperatively for 2 days, then shifted to the ward. The patient recovered gradually and suffered no complications postoperatively. She was discharged home on day 8 postop on laxatives.

The patient demonstrated good recovery with no complications in her first follow-up visit. However, in her subsequent follow-up visits, she complained of hard stoma output and abdominal distention. She was advised to continue on regular laxatives with diet habit modifications. Nine months later, she complained of significant constipation despite being on high-dose laxatives. Additionally, she reported progressive dysphagia and regurgitation to both solids and liquids. Given the patient condition and symptoms, the plan was to postpone colostomy reversal and to do further investigations to assess for possible GI motility disorders.

**Figure 3.**
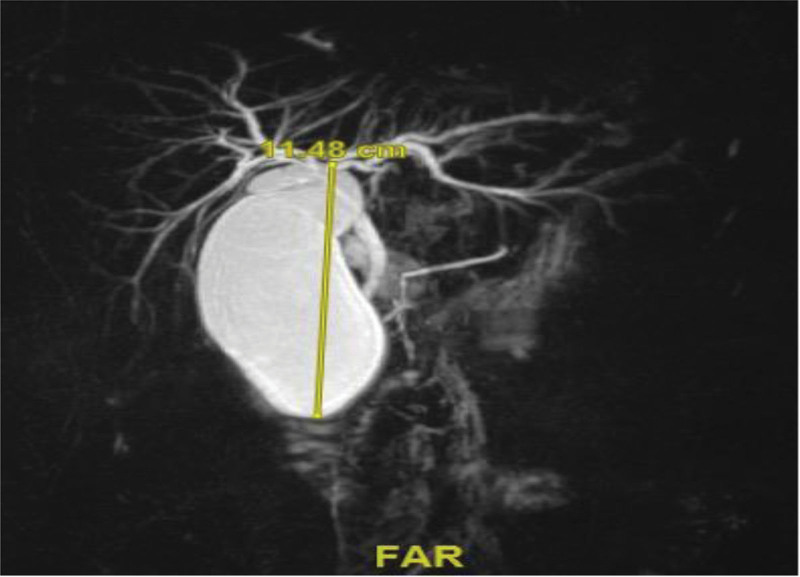
Magnetic resonance cholangiopancreatography (MRCP) image showed markedly dilated gallbladder with no apparent stricture.

The patient’s work-up included upper GI endoscopy that showed partial loss of duodenal folds and villous architecture, biopsies were sent, and the result was negative for celiac disease. Additionally, esophageal manometry revealed high integrated relaxation pressure with distal latency < 4.5 seconds in > 20% of swallows, suggestive of Achalasia type 3 per Chicago Classification. Colonoscopy was repeated with aggressive bowel preparation, which showed mild colitis. Biopsies revealed mild chronic colitis with focal crypt abscess formation. The gallbladder and biliary system were investigated with Magnetic resonance cholangiopancreatography (MRCP) and showed markedly dilated gallbladder (gallbladder hydrops) and mild intrahepatic bile duct dilatation, with no visualized strictures or impacted stones (Fig. 3). A nuclear study assessed stomach motility, which showed a mild delay in gastric emptying.

The patient and her husband were counseled about her medical condition and planned to proceed with redo-Heller myotomy and reverse Hartmann after proper constipation control. The risk and benefits were explained to the patient. She decided to proceed with both procedures at the same time.

Subsequently, the patient underwent a robotic redo of Heller myotomy and Dor fundoplication and reversed Hartmann procedure. The surgery was uneventful with no complications. The patient was later discharged home on postop day 4 on regular laxatives. She was seen in the surgical clinic after discharge, where she was well, tolerating diet, and passing well-formed bowel movements. The patient continued her follow-up with the GI motility clinic for further medical optimization.

## 3. Discussion and Conclusion

The gastrointestinal motility disorder can present as an isolated single organ disease or diffuse with more than 1 organ involvement, such as achalasia, intestinal pseudo-obstruction, gastroparesis, and colonic inertia.^[3]^ In our case, the patient initially presented with achalasia that progressed several years later to diffuse GI motility disorder with the presentation of acute life-threatening consequences of chronic constipation.

The most prevalent motility disorder of the esophagus is achalasia, which typically presents as dysphagia, regurgitation, and chest pain. Esophageal manometry will demonstrate insufficient relaxation of the lower esophageal sphincter and proximal aperistalsis.^[4]^ Chicago Classification is used to interpret the high-resolution manometric findings for esophageal motility disorders, and a normal integrated relaxation pressure is <15. Also, if the Distal Latency result is shorter than 4.5 seconds, it is considered premature or spastic. On the other hand, it is considered normal when it’s longer than 4.5 .^[5]^ Treatment options include botulinum toxin injection, pneumatic dilation, and Heller myotomy.^[4]^ Our patient was managed by Heller myotomy 15 years ago. However, recently she started to complain of progressive dysphagia and regurgitation, which necessitated redo-Heller myotomy.

Constipation is one of the most prevalent gastrointestinal illnesses, accounting for 15 to 20% of all gastrointestinal illnesses.^[6]^ Recent evidence suggests that it is more frequent in women, and it becomes more prevalent as the population ages.^[7]^ There are 2 types of chronic constipation, primary and secondary constipation. Primary constipation is further classified as normal transit, slow transit, and defecatory disorders. Secondary constipation is usually caused by medications, diet, anatomical alteration, neurological conditions, metabolic conditions, and rarely colon cancer.^[8,9]^ Chronically constipated patients face a tremendously negative effect on their quality of life. A systematic review conducted by Belsey et al showed that the quality of life for patients with constipation is significantly impaired compared to other chronic conditions. The most notable effect was found in the emotional and mental aspects.^[10]^

Chronic constipation can cause fecal impaction in 50% of the cases, and it accounts for 3.2% of the causes of colonic perforation.^[11,12]^ The pathophysiology behind the perforation is attributed to the rise in the intraluminal pressure caused by the fecaloma, which will result in the development of ischemia and colitis. This inflammatory reaction will lead to ulceration and a subsequent stercoral perforation.^[13]^ The sigmoid and rectosigmoid colon is the most likely affected segments due to several possible explanations; narrow diameter of the colon, solid consistency of the feces, and insufficient vascularization, particularly in the antimesenteric border.^[14]^ The perforation was due to a large fecaloma impaction resulting in a perforated mid-sigmoid at the antimesenteric border.

In any patient presenting with severe chronic constipation that results in bowel perforation, investigations for mechanical causes should be done. When this possibility is ruled out, gastrointestinal motility disorders should be assessed.^[15]^ Motility studies of the esophagus, stomach, small bowel, colon, and biliary system are needed to confirm the diagnosis. Gastric emptying scintigraphy is performed to evaluate gastric emptying in patients with symptoms and signs suggestive of impaired gastric emptying.^[16]^ To evaluate colonic motility, colonic transit time is used to determine the severity and the response to therapy using radiopaque markers or barium suspension.^[17,18]^ Hepatobiliary iminodiacetic acid (HIDA) scan is a nuclear study that uses radiotracers to evaluate the function of the biliary system and liver.^[19]^

The management goal in chronically constipated patients is to improve and regain normal bowel habits.^[8]^ Treatment options include lifestyle and diet changes, biofeedback therapy, and pharmacological therapy. Failure of nonsurgical treatment is sometimes seen, and surgical options should be considered in those patients. Partial, segmental, and occasionally total colectomy is performed in severe, rare cases of colonic inertia or colonic neuropathy.^[6]^ Other surgical interventions include the formation of an ileostomy, which is an acceptable choice when total colectomy is not suitable.^[7]^ As has been previously reported in the literature, when performing a total colectomy, there is an increased risk of mortality and causing damage to surrounding structures (e.g., ureter, duodenum) compared to another option, the colonic bypass surgery. Colonic bypass is a less aggressive approach offered to patients who rejected the total colectomy with ileorectal anastomosis.^[20]^

In our case, the patient presented with stercoral perforation. We managed the patient with segmental colon resection (Hartman procedure) followed by anastomosis at a later stage. Postoperatively, constipation was controlled well with regular laxatives. A recent case reported by Chen et al (2021) also supports performing Hartman procedure to manage stercoral perforation, which is a risk in chronically constipated patients.^[21]^ Furthermore, in emergency cases, Hartmann procedure is a safe approach.^[22]^ Fortunately, she did not require any further surgical intervention.

In conclusion, gastrointestinal motility disorders can involve any part of the GI tract. Initial investigations should be directed to rule out mechanical and organic causes. GI motility disorders can present with life-threatening complications of colonic perforation. Early recognition and prompt treatment of GI motility disorders are essential to avoid severe complications.

## Author contributions

Conceptualization: Ahmed Alburakan

Data curation: Sulaiman Alshammari

Methodology: Ahmed Alburakan, Saleh Alnasser, Thamer Nouh

Project administration: Ahmed Alburakan

Resources: Ahmed Alburakan, Saleh Alnasser, Thamer Nouh

Supervision: Ahmed Alburakan

Writing - original draft: Aljoharah Alshunaifi, Razan AlRabah, Sulaiman Alshammari

Writing - review & editing: Aljoharah Alshunaifi, Razan AlRabah, Sulaiman Alshammari,

Ahmed Alburakan
